# Complete mitochondrial genomes of three reef forming *Acropora* corals (Acroporidae, Scleractinia) from Chagos Archipelago, Indian Ocean

**DOI:** 10.3897/BDJ.9.e72762

**Published:** 2021-09-30

**Authors:** Luigi Colin, Chris Yesson, Catherine E. I. Head

**Affiliations:** 1 Institute of Zoology, Zoological Society of London, Regent's Park, NW1 4RY, London, United Kingdom Institute of Zoology, Zoological Society of London, Regent's Park, NW1 4RY London United Kingdom; 2 Department of Zoology, University of Oxford, John Krebs Field Station, Wytham, OX2 8JQ, Oxford, United Kingdom Department of Zoology, University of Oxford, John Krebs Field Station, Wytham, OX2 8JQ Oxford United Kingdom; 3 St Peter’s College, New Inn Hall Street, OX1 2DL, Oxford, United Kingdom St Peter’s College, New Inn Hall Street, OX1 2DL Oxford United Kingdom

**Keywords:** mitochondrial genome, Acropora, Chagos Archipelago

## Abstract

We present the first mitochondrial genomes from Chagos Archipelago, Indian Ocean, of three putative species of reef forming Acropora (Acropora aff. tenuis, Acropora aff.cytherea and Acropora aff. orbicularis). The circular genome consists respectively of 18,334 bp, 18,353 bp and 18,584 bp. All mitochondrial genomes recovered comprise 13 protein-coding genes, two transfer RNA genes and two ribosomal RNA genes, with an overall GC content ranging from 37.9% to 38.0%. These new genomic data contribute to our increased understanding of genus *Acropora* and its species boundaries, ultimately aiding species monitoring and conservation efforts.

## Introduction

The genus *Acropora* (Scleractinia, Acroporidae) is a widespread coral, spanning the Indian and Pacific Oceans and the Caribbean Sea (Van Oppen et al. 2001, Veron et al. 2020) and is one of the major reef builders in warm water ecosystems (Fukami et al. 2000). Warm water reef-building corals create some of the most biodiverse ecosystems on the planet and have been estimated to support 830,000 species of multi-cellular plants and animals worldwide (Bellwood and Hughes 2001, Mora et al. 2008, Mora et al. 2011, Fisher et al. 2015), providing a variety of habitats for fish, invertebrates and other taxa in shallow tropical seas (Bellwood and Hughes 2001). Despite the importance of reef-building corals, species' boundaries are considered somewhat blurry and previous investigations on the *Acropora* genus show that many morphological species in this genus do not correspond to genetically distinct evolutionary units, with examples of intraspecific geographic differences in morphology as large as differences between species (Van Oppen et al. 2001). The Chagos Archipelago’s *Acropora* and *Porites* dominated reefs (Head et al. 2019) constitute around 2.5% of the world’s reefs (Sheppard 1999) and are a potential “stepping stone” for transoceanic species dispersal (Sheppard et al. 2012), so it is a key geographical location for further research.

Systematic research is defined as an interactive process in which taxa are defined or redefined by synthesis of all available information from biological, molecular and other relevant areas of science (WILSON 1985, Wallace and Willis 1994). Systematics attempts to keep pace with developments in these fields so that the most appropriate taxonomic interpretation will facilitate the greatest possible accuracy of research and experimental design (Wallace and Willis 1994). Despite a long history of taxonomic work, Scleractinia systematics is still largely unresolved (Richards et al. 2016) and species identifications in this group have been known to be problematic for more than four decades (Randall 1981, Wallace and Willis 1994). Further issues for *Acropora* include cases of shared recent ancestry and introgression of loci from ongoing hybridisation (Van Oppen et al. 2001, Willis et al. 2006, Richards et al. 2013, Richards et al. 2016). Species boundaries, currently applied to *Acropora*, do not stand up to scrutiny; this may in part be methodological, but current species boundaries are also confounded by characteristics of *Acropora*, such as morphological variability and hybridisation potential (Wallace and Willis 1994). The existence of morphologically cryptic species within recognised “species” of stony corals (Richards et al. 2016), together with evidence of strong and recurring regional genetic differentiation corresponding to the separation of the Indian and Pacific Ocean in *Acropora* (Richards et al. 2016), support the need for improved sampling across geographically distinct populations.

Mitochondrial DNA (mtDNA) has been used in numerous applications in the past 20 years, ranging from species delimitation (Paz-García et al. 2016) – usually focused on the mitochondrially encoded cytochrome c oxidase I (MT-CO1 or CO1 or COX1) (Hebert et al. 2003) – to phylogeny and molecular evolutionary studies (Curole and Kocher 1999, Niu et al. 2020). The mitochondrial genome plays a significant role in studies of phylogenetic reconstruction (Fukami and Knowlton 2005, Arrigoni et al. 2016, Capel et al. 2016, Terraneo et al. 2018, Terraneo et al. 2018), mostly due to a general consensus in the gene order and infrequent mitochondrial genome rearrangements across scleractinians corals (van Oppen et al. 2002, Lin et al. 2012). It typically includes 13 oxidative phosphorylation (OXPHOS) related genes, two rRNAs that encode the two subunits of mitochondrial ribosomes and an array of tRNAs used for translation within the organelle (Niu et al. 2020). Studying the mitochondrial genome could help to further explore Scleractinia’s evolutionary process and clarify the evolutionary relationship between Scleractinia and other Hexacorallia members (Lin et al. 2012).

## Data resources

### Data Availability Statement

The genome sequence data that support the findings of this study are openly available in GenBank of NCBI at https://www.ncbi.nlm.nih.gov/ under the accession no. MW773216 - MW773217 - MW773218. The associated **BioProject**, **SRA** and **Bio-Sample** numbers are listed in Table 1.

## Material and methods

### Study Area and Collection

**Figure 1.**
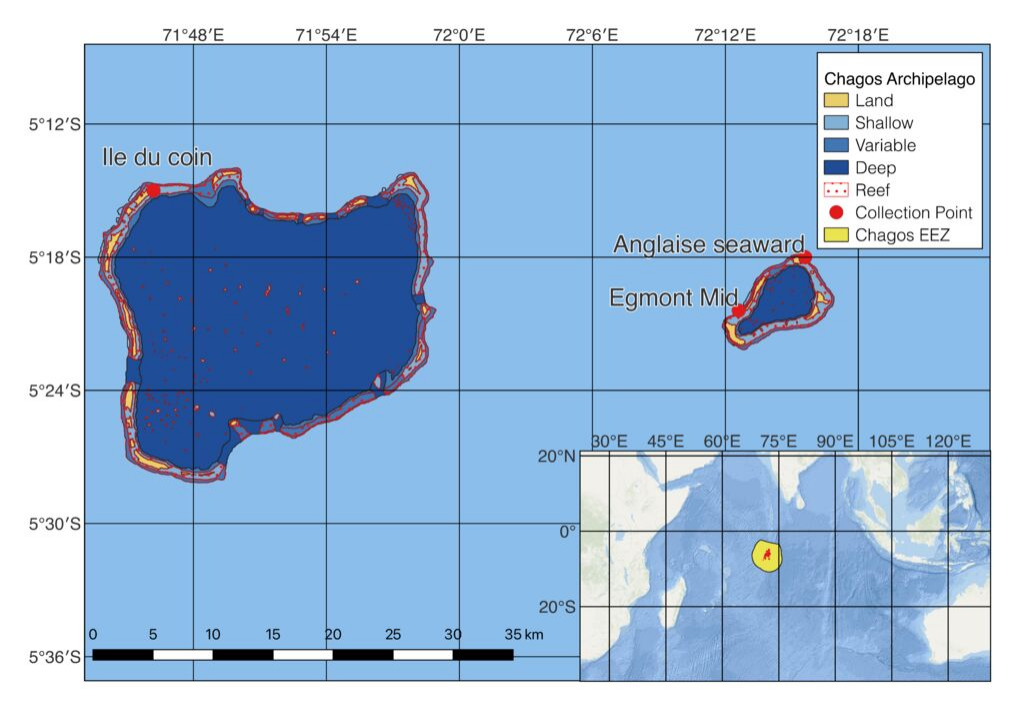
Map of Peros Banhos and Salomon Islands with the three collection sites accompanied by location of the larger Chagos Archipelago. The samples were collected respectively at Egmont Mid for Acroporaaff.tenuis, Ile Anglaise seaward for Acroporaaff.cytherea and Ile du coin Acroporaaff.orbicularis.

Samples were collected from three sites across Chagos Archipelago (Egmont Mid "-5.34, 72.21", Ile Anglaise seaward "-5.30, 72.26", Ile du coin "-5.25, 71.77" - Fig. 1) as part of the 2018 Chagos Reef 1 Expedition (CITES permits no. 567238/01 - 567238/02).

A fragment of coral 2-3 cm^2^ in size, containing one or more healthy-looking polyps, was collected during SCUBA surveys and a corresponding photo of the colony was taken (Suppl. materials 1, 2, 3). In order to account for possible clonality, samples were collected at least one metre distance from each other. Upon returning to the ship, the samples were immediately placed in single vials in ethanol (+95%) and labelled with a unique identifier, in addition to collection date and location. The samples were then stored at -20°C until extraction.

Sample identification was performed in the field by eye by a coral expert (Dr Catherine Head). Identification was confirmed by Dr Tom Bridge, Senior Curator of Corals at the Queensland Museum Network (QMN), based on morphology from the field photos alongside phylogenetic methods (ultraconserved elements (UCEs) via hybrid capture (Quattrini et al. 2017, Zhang et al. 2019, Cowman et al. 2020))

### DNA extraction

Total genomic DNA (gDNA) was extracted from four 0.5 to 2 cm coral fragments (Samples deposited at The Natural History Museum, London, UK, Table 1). The extraction followed a modified version of the manufacturer protocol for the DNeasy PowerSoil Pro Kit from Qiagen© (Protocols.io: dx.doi.org/10.17504/protocols.io.bww6pfhe). Following quantification of double-stranded DNA with a Qubit fluorometer 2.0 (Invitrogen, Waltham, MA), three separate indexed libraries were constructed with the gDNA by ligation kit (Oxford Nanopore Technologies) and subsequently pooled, prior to sequencing on a MinION sequencer (ligation kit: SQK-LSK109; indexes: EXP-NBD104; flowcell: R9 FLO-MIN106D).

### Mitogenome assembly

Reads were demultiplexed and adapter trimmed using Porechop v.0.2.4 (https://github.com/rrwick/Porechop); subsequently, reads were mapped with Geneious mapper (Geneious Prime 2021.1.1.) to existing GenBank (Benson 2000) reference mitochondrial genomes (seven Acropora species: NC_003522, NC_022824, NC_022826, NC_022828, NC_022829, NC_022830 and NC_022831) and mapped reads were selected and de novo assembly conducted using CANU v.2.1.1 (Koren et al. 2017). The assemblies were then imported into the NanoGalaxy public server (de Koning et al. 2020) and polished using medaka (Oxford Nanopore Technologies Ltd. 2018 -Lu et al. 2016). Initial quality and annotation check for each assembly was performed with Quast (Gurevich et al. 2013, Mikheenko et al. 2015, Mikheenko et al. 2016, Mikheenko et al. 2018).

**Figure 2.**
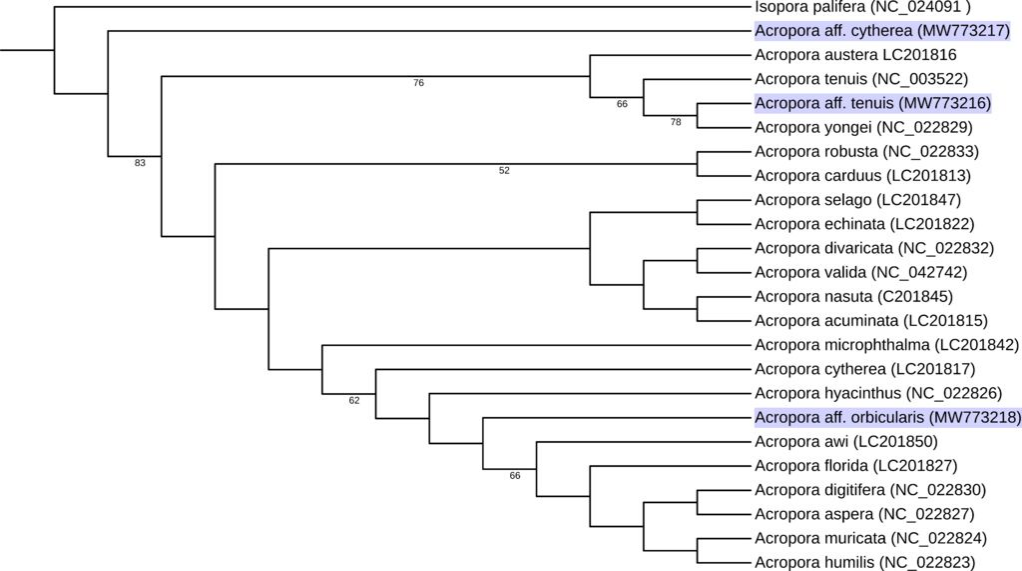
Maximum Likelihood phylogeny from analysis of concatenated protein-coding genes. Specimens from Chagos Archipelago annotated in blue. Bootstrap support numbers shown at nodes with > 50% support. GenBank accession numbers in parentheses. Outgroup - *Isoporapalifera* NC_024091.

Subsequently, feature annotations were transferred in Geneious Prime and verified by comparison with alignments of coding regions with the above-mentioned references from GenBank. Inside coding regions, extra bases within a repeated seqeunce were manually removed if they caused a clear and significant frame-shift. Amino acid sequences of 13 concatenated protein-coding genes from our assemblies, together with 20 reference sequences were aligned using Geneious. Aligned sequences were uploaded to the European Galaxy server (Afgan et al. 2018) and subjected to phylogenetic analysis using IQ-TREE, with a 1000 non-parametric bootstrap, automatic model selection and default settings (Minh et al. 2013, Nguyen et al. 2015, Kalyaanamoorthy et al. 2017) and automatic amino acid substitution model selection (Fig. 2). Management, visualisation and annotation of the tree was done through iTOL v.6 (https://itol.embl.de - Letunic and Bork 2021).

## Results

We determined the mitochondrial genome of *Acropora* aff. *tenuis, Acropora* aff. *cytherea, Acropora* aff.orbicularis to be respectively 18334 bp, 18353 bp and 18584 bp in length. The sequences are deposited in GenBank under accession no. MW773216, MW773217 and MW773218. The mitochondrial genome codes for 17 genes: 13 protein-coding genes, two tRNA genes and the large 16S and small 12S rRNA genes. Gene order follows an identical pattern to those of other *Acropora* mitochondrial genomes. Start and stop codons are reported in Table 2. The overall GC content is 37.9%, 38.0% and 38.0%, respectively, with an overall GC skew of 0. 0.28 and AT skew of 0.19. Nucleotide composition of the entire mitochondrial genome is: Acroporaaff.tenuis - A = 4,602 (25.1%), C = 2,514 (13.7%), G = 4,435 (24.2%), T = 6,783 (37.0%); Acroporaaff.cytherea - A = 3,280 (23.8%), C = 1,891 (13.7%), G = 3,305 (24.0%), T = 5,309 (38.5%); Acroporaaff.orbicularis - A = 4,645 (25.0%), C = 2,551 (13.7%), G = 4,516 (24.3%), T = 6,872 (37.0%). Where available, sequences of species matching the names of our tentative 'aff' assignments were included in the phylogeny; however, our samples did not group with these taxa (Fig. 2), supporting the tentative nature of these identifications and strengthening the case that these are distinct, possibly morphologically cryptic, species. These new genomic data contribute to our increased understanding of the phylogenetic history and mitochondrial evolution patterns in the *Acropora* genus, ultimately aiding species monitoring and conservation efforts.

## Supplementary Material

B8AB54D2-9A7E-512B-AA2E-5DD2A8D59A1C 10.3897/BDJ.9.e72762.suppl1 Supplementary material 1 Field photo of coral colony - Acroporaaff.tenuis Data type images File: oo_592369.jpg https://binary.pensoft.net/file/592369 Dr Catherine Head

A4C7CD32-E779-54ED-82F7-0E3132DC2F38 10.3897/BDJ.9.e72762.suppl2 Supplementary material 2 Field photo of coral colony - Acroporaaff.cytherea Data type images File: oo_592373.jpg https://binary.pensoft.net/file/592373 Dr Catherine Head

E0FD22DF-25D3-5D8E-A292-C0FE6B29E870 10.3897/BDJ.9.e72762.suppl3 Supplementary material 3 Field photo of coral colony - Acroporaaff.orbicularis Data type images File: oo_592365.jpg https://binary.pensoft.net/file/592365 Dr Catherine Head

## Figures and Tables

**Table 1.** Data Availability Table

**Accession no.**	**BioProject**	**SRA**	**BioSample**	**Sample**	Museum accession no.*
MW773216	PRJNA720633	SRR14216029	SAMN18673282	Acroporaaff.tenuis	NHMUK 2021.1
MW773217	PRJNA720633	SRR14216028	SAMN18673283	Acroporaaff.cytherea	NHMUK 2021.2
MW773218	PRJNA720633	SRR14216027	SAMN18673284	Acroporaaff.orbicularis	NHMUK 2021.3

*All sample deposited at The Natural History Museum, London (UK)

**Table 2.** Start/stop codon of all protein-coding genes

	**COX1**	**ATP8**	**ND3**	**ND4L**	**COX2**	**COX3**	**ND4**	**ATP6**	**ND6**	**ND2**	**CYTB**	**ND1**	**ND5**
Start codon	ATG	ATG	GTG	GTG	ATG	GTG	GTG	ATG	ATA	ATG	ATG	GTG	GTG
Stop codon	TAA	TAG	TAG	TAA	TAG	TAG	TAG	TAG	TAA	TAA	TAG	TAA	TAG
