# Supplementary figures and images for: Complete mitochondrial genomes of three reef forming Acropora corals (Acroporidae, Scleractinia) from Chagos Archipelago, Indian Ocean
Source: Biodivers Data J. 2021 Sep 30;9:e72762. doi: 10.3897/BDJ.9.e72762 (PMC8497460; doi:10.3897/BDJ.9.e72762)

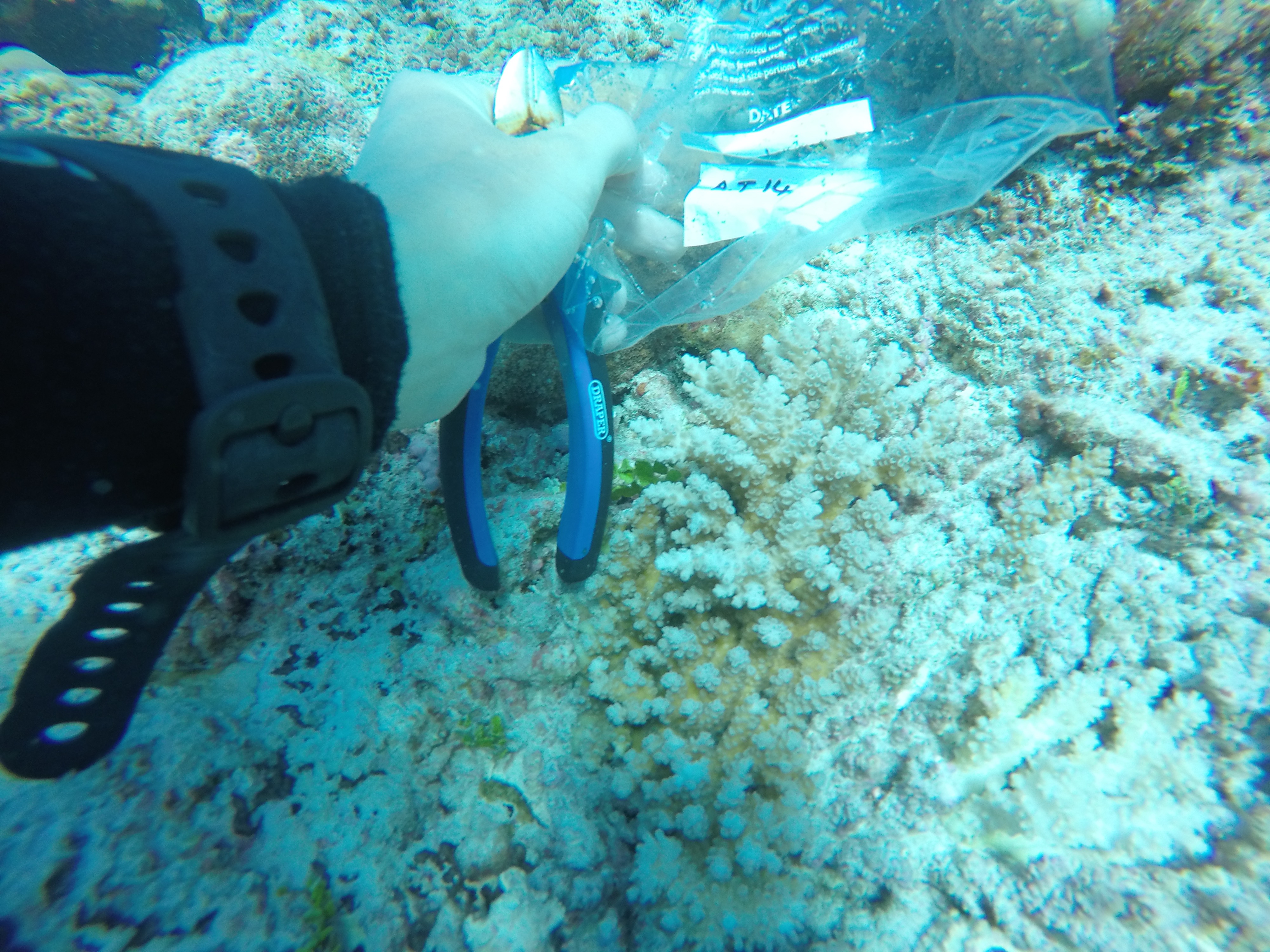

Supplement: Supplementary material 1 — Field photo of coral colony - Acroporaaff.tenuis [file bdj-09-e72762-s001.jpg]

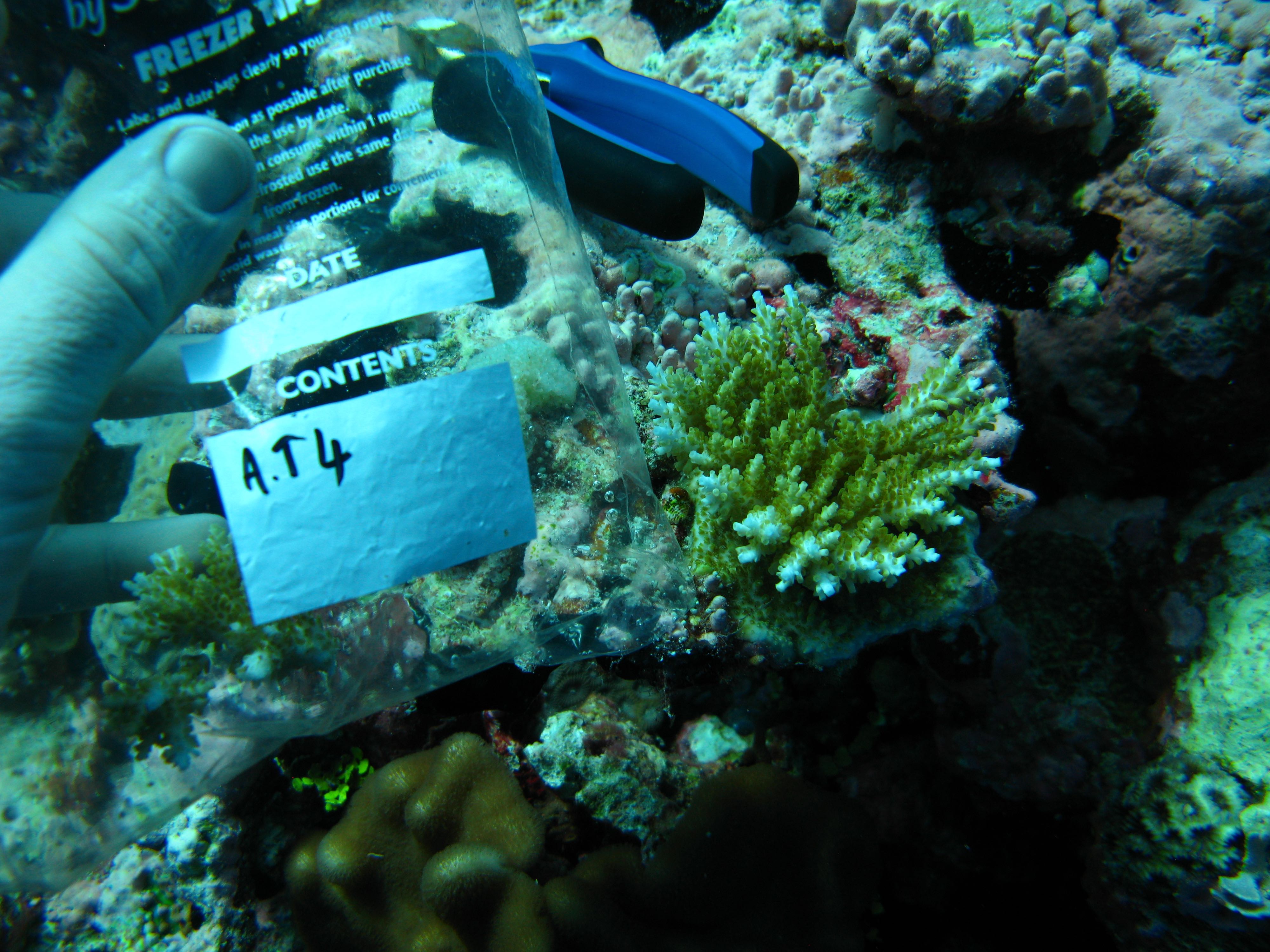

Supplement: Supplementary material 2 — Field photo of coral colony - Acroporaaff.cytherea [file bdj-09-e72762-s002.jpg]

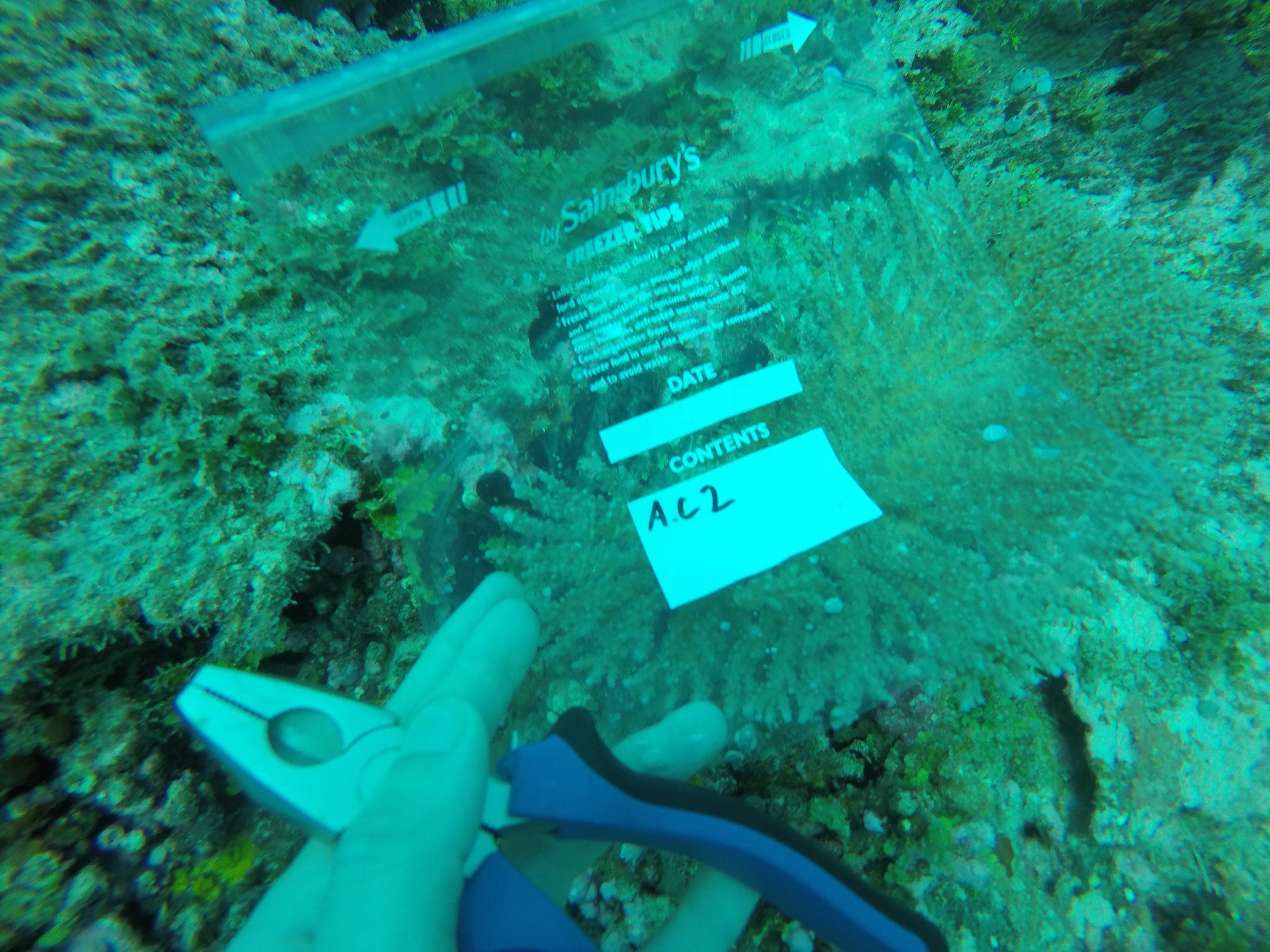

Supplement: Supplementary material 3 — Field photo of coral colony - Acroporaaff.orbicularis [file bdj-09-e72762-s003.jpg]
